# The temporal program of peripheral blood gene expression in the response of nonhuman primates to Ebola hemorrhagic fever

**DOI:** 10.1186/gb-2007-8-8-r174

**Published:** 2007-08-28

**Authors:** Kathleen H Rubins, Lisa E Hensley, Victoria Wahl-Jensen, Kathleen M Daddario DiCaprio, Howard A Young, Douglas S Reed, Peter B Jahrling, Patrick O Brown, David A Relman, Thomas W Geisbert

**Affiliations:** 1 Department of Microbiology and Immunology, 299 Campus Dr., Stanford University School of Medicine, Stanford, California 94305, USA; 2 Department of Biochemistry, 279 Campus Dr., Stanford University School of Medicine, Stanford, California 94305, USA; 3 Whitehead Institute for Biomedical Research, Nine Cambridge Center, Cambridge, Massachusetts 02142, USA; 4 US Army Medical Research Institute of Infectious Diseases, 1425 Porter St., Fort Detrick, Maryland 21702-5011, USA; 5 National Cancer Institute - Frederick, 1050 Boyles St., Frederick, Maryland 21702, USA; 6 Howard Hughes Medical Institute, 279 Campus Dr., Stanford University School of Medicine, Stanford, California 94305, USA; 7 Department of Medicine, 300 Pasteur Dr., Stanford University School of Medicine, Stanford, California 94305, USA; 8 Veterans Affairs Palo Alto Health Care System, 3801 Miranda Ave., Palo Alto, California 94304, USA

## Abstract

**Background:**

Infection with Ebola virus (EBOV) causes a fulminant and often fatal hemorrhagic fever. In order to improve our understanding of EBOV pathogenesis and EBOV-host interactions, we examined the molecular features of EBOV infection *in vivo*.

**Results:**

Using high-density cDNA microarrays, we analyzed genome-wide host expression patterns in sequential blood samples from nonhuman primates infected with EBOV. The temporal program of gene expression was strikingly similar between animals. Of particular interest were features of the data that reflect the interferon response, cytokine signaling, and apoptosis. Transcript levels for tumor necrosis factor-α converting enzyme (TACE)/α-disintegrin and metalloproteinase (ADAM)-17 increased during days 4 to 6 after infection. In addition, the serum concentration of cleaved Ebola glycoprotein (GP_2 delta_) was elevated in late-stage EBOV infected animals. Of note, we were able to detect changes in gene expression of more than 300 genes before symptoms appeared.

**Conclusion:**

These results provide the first genome-wide *ex vivo *analysis of the host response to systemic filovirus infection and disease. These data may elucidate mechanisms of viral pathogenesis and host defense, and may suggest targets for diagnostic and therapeutic development.

## Abstract 2

Primate blood cells were analysed for changes in global gene expression patterns at several time points following infection with Ebola virus, providing insights into potential mechanisms of viral pathogenesis and host defense.

## Background

Ebola virus causes severe and often lethal hemorrhagic fever in humans and nonhuman primates. Ebola virus (EBOV) is one of two genera that comprise the family Filoviridae. The EBOV genus consists of four distinct species: Ivory Coast Ebola virus, Reston Ebola virus, Sudan Ebola virus, and Zaire Ebola virus (ZEBOV) [1]. Sudan Ebola virus and ZEBOV have been associated with human disease outbreaks in Central Africa, with case fatality rates averaging about 50% for Sudan Ebola virus and ranging from 75% to 90% for ZEBOV [2]. Although Reston Ebola virus is highly lethal in nonhuman primates [3,4], the few data available suggest that it is nonpathogenic in humans [5]. The pathogenic potential of Ivory Coast Ebola virus is unclear because there has only been a single confirmed nonfatal human case [6] and a second suspected nonfatal case [7]. In addition to natural outbreaks, EBOV is an important concern as a potential biologic threat agent of deliberate use because these viruses have low infectious doses and clear potential for dissemination by aerosol route [8]. Currently, there are no approved preventive vaccines or postexposure treatments for EBOV hemorrhagic fever, but recent advances have led to the development of several candidate therapeutics and vaccines for EBOV [9-11].

The mechanisms of EBOV pathogenesis are only partially understood, but dysregulation of normal host immune responses (including destruction of lymphocytes [2] and increases in levels of circulating proinflammatory cytokines [12]) is thought to play a major role. Several animal models of EBOV hemorrhagic fever have been developed, notably a cynomolgus macaque (*Macaca fascicularis*) model [13,14], which closely resembles human infection [2,15]. ZEBOV infection in cynomolgus macaques results in uniform lethality at days 6 to 7 after infection [16-19].

The majority of studies conducted in nonhuman primates have focused on end-point examination when animals are in the final stages of disease, and have restricted their analyses to small numbers of cytokines or mRNA transcripts. cDNA microarrays have been used by our group to study mechanisms of viral pathogenesis in a nonhuman primate model of an agent, albeit unrelated, that also causes overwhelming, systemic infection [20,21]. In order to understand better the early events in EBOV pathogenesis, we examined global changes in gene transcript abundance, using cDNA microarrays, in sequential blood samples from 21 cynomolgus macaques over the entire time course of ZEBOV infection.

## Results

### Dataset overview

**Figure 1:**
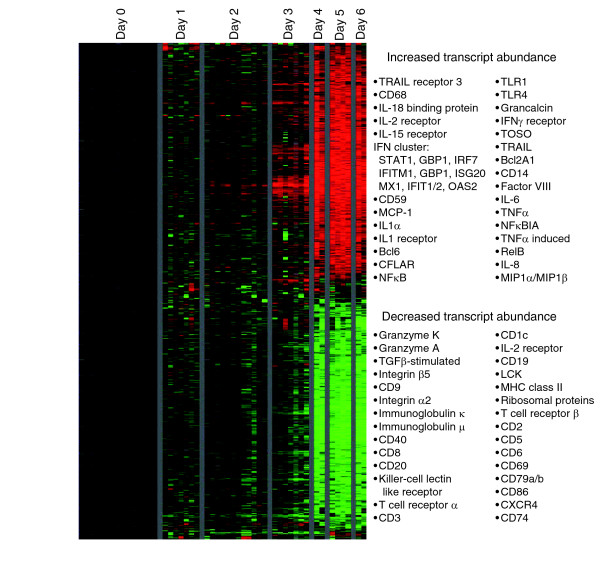
Overview of gene expression in peripheral blood mononuclear cells from Ebola infected macaques. A total of 3,670 elements (1,832 named genes) exhibited a threefold change or greater in mRNA abundance from at least three different arrays. The data for these 3,670 elements were hierarchically clustered [67]. Data from individual elements or genes are represented as a single row, and samples from individual monkeys at different days after infection are shown as columns. Red and green colors denote expression levels greater or less, respectively, than baseline values (average of two to three samples taken at day -1 and day -6 before inoculation). The intensity of the color reflects the magnitude of the change from baseline.

We characterized the host gene expression program in peripheral blood mononuclear cells (PBMCs) of cynomolgus macaques during a temporal survey of ZEBOV infection. The dataset from these experiments comprises about 3.2 million measurements of transcript abundance in a total of 65 blood samples from 21 animals using 85 DNA microarrays. Additional data file 1 shows animal numbers corresponding to blood samples. Samples are arranged in the table order (namely, days 0 to 6 after infection), from right to left, in all figures. The bleed schedule is provided in Additional data file 2. Figure 1 provides an overview of the temporal changes in gene expression patterns in PBMCs. The gene expression program exhibits surprisingly consistent patterns of temporal regulation among all animals sampled, with very few changes with respect to baseline evident at days 1 and 2 after infection, followed by dramatic and widespread changes at days 4 to 6 after infection. During this latter phase there were changes of at least threefold in the relative abundance of transcripts for more than 3,760 elements (1,832 unique named genes; Figure 1 and Additional data file 3). The average pair-wise correlation of the expression profiles of these 3,760 elements (1,832 named genes) between different animals at days 4, 5, and 6 after infection was 0.85, demonstrating the consistency of host response in this model. In comparison, using the same criteria the average pair-wise correlation of the transcript abundance patterns between animals in a cynomolgus macaque model of smallpox infection was 0.55 over 2,387 elements for the same time frame [20].

### Cytokine response and innate immune activation

**Figure 2:**
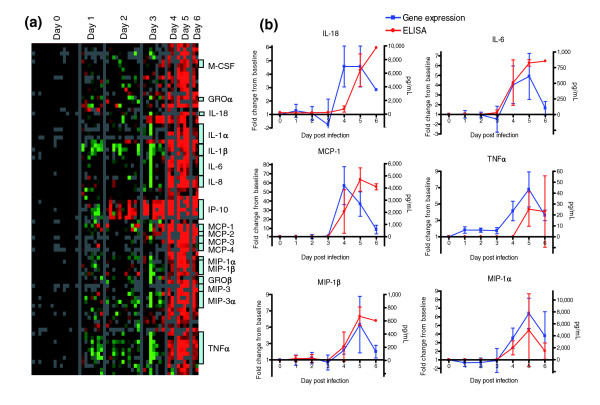
Cytokine gene expression. **(a) **A list of all cytokines and chemokines (as defined by Gene Ontology annotation) was used to extract gene expression data. Genes with at least a 2.5-fold change from baseline in at least three arrays are displayed. **(b) **Transcript levels of cytokine mRNA in peripheral blood mononuclear cells and ELISAs for detection of soluble cytokines in the serum. IL, interleukin; MCP, monocyte chemoattractant protein; MIP, macrophage inflammatory protein; TNF, tumor necrosis factor.

A significant increase in cytokine and chemokine transcripts was observed at days 4 to 6 after infection (Figure 2a). Transcripts encoding the proinflammatory cytokines IL-1β, IL-6, IL-8, and tumor necrosis factor (TNF)-α were markedly increased in late-stage animals (average fold increase at day 5 after infection: IL-1β, 3.9; IL-6, 4.3; IL-8, 11.3; and TNF-α, 5.2; Figure 2b). In addition, several chemokines (macrophage inflammatory protein [MIP]-1α, MIP-1β, growth related oncogene-α, growth related oncogene-β, monocyte chemoattractant protein [MCP]-1, MCP-2, MCP-3, and MCP-4) exhibited increased transcript levels at days 4 to 6 after infection in all animals (Figure 2a). Transcripts for several other cytokines (IL-2, IL-4, IL-10, and IL-12) were detected on the array, but their levels did not change significantly during the course of infection. We measured levels of soluble cytokines by ELISA. All measured cytokines for which we also have gene expression data are shown in Figure 2b. IL-6 and MCP-1 showed marked increases by day 4 after infection; and MIP-1α and MIP-1β exhibited moderate increases on day 4, coinciding with gene expression data. By day 5 these four cytokines were elevated, and there was also an increase in TNF-α and IL-18 in serum. The ELISA data closely parallel the microarray mRNA expression data.

**Figure 3:**
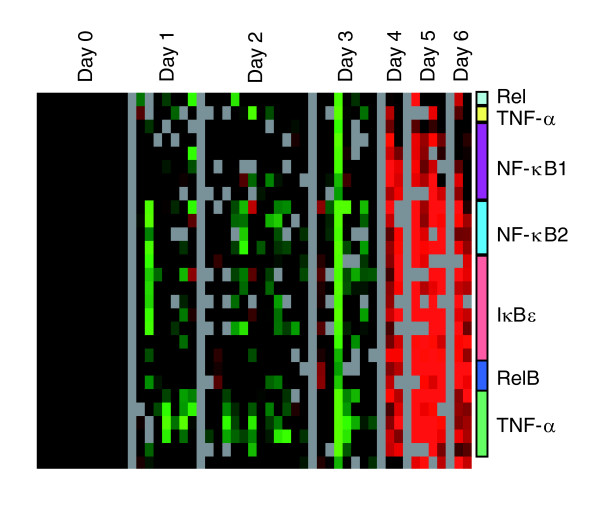
Tumor necrosis factor-α/nuclear factor-κB response. The set of genes representing the tumor necrosis factor (TNF)-α/nuclear factor-κB (NF-κB) regulon present in previously published lipopolysaccharide stimulation data [22] was extracted from the dataset and hierarchically clustered. Colored bars represent multiple clones on the array for a given gene.

We previously identified a set of genes representing the TNF-α/nuclear factor-κB (NF-κB) B regulon as a prominent feature of the PBMC response to bacterial lipopolysaccharide [22]. We extracted these genes from the ZEBOV dataset and saw marked induction in transcripts regulated by TNF-α/NF-κB (Figure 3).

### Apoptosis

**Figure 4:**
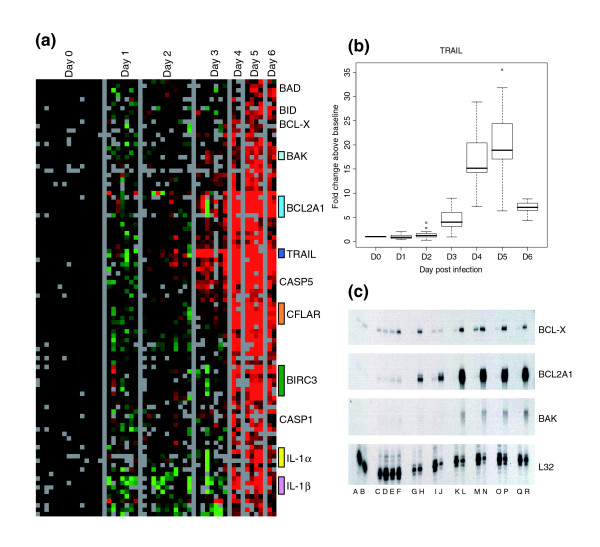
Apoptosis-related genes. **(a) **The set of apoptosis-related genes (as defined by Gene Ontology annotation) was used to extract gene expression data. Genes with at least a 2.5-fold change from baseline in at least two arrays are displayed. **(b) **Transcript levels for tumor necrosis factor (ligand) superfamily, member 10 (TNFSF10/TRAIL) at various times after infection. **(c) **Transcript levels of apoptosis-related genes, as determined by RNAase protection assays at day 0 after infection (lanes A, C, G, I, K, M, O, and Q), day 1 after infection (lanes B and D), day 2 after infection (lane E), day 3 after infection (lane F), day 4 after infection (lanes H and J), day 5 after infection (lanes L, N, and P), day 6 after infection (lane R). Colored bars represent multiple clones on the array for a given gene.

Lymphocyte apoptosis in the lymph node and spleen has previously been identified as a hallmark of ZEBOV infection and a potential contributor to pathogenesis [23-25]. In order to determine whether we could also detect evidence of apoptosis in circulating PBMCs we examined the dataset for genes with Gene Ontology (GO) annotation for involvement in apoptosis (pro-apoptotic or anti-apoptotic). Transcripts of a set of genes that play a role in regulating apoptosis increased on days 4 to 6 after infection (Figure 4a). These genes included Bcl-2 family members and interacting proteins: BCL2-antagonist of cell death, BH3 interacting domain death agonist, BCL2-like 1 (BCL2L1/BCL-X), BCL2-related protein A1, TNF superfamily member 10 (also known as TNF related apoptosis inducing ligand [TRAIL]), caspase-5, caspase-8, FADD (Fas-associated death domain protein)-like apoptosis regulator, caspase 1 apoptosis-related cysteine peptidase/IL-1β convertase, IL-1β, and IL-1α. TRAIL transcript abundance increased as much as 35-fold above background at day 5 in some animals, with average expression being 19.4-fold above baseline (Figure 4b). We confirmed induction of several of these transcripts (BCL-X, BCL2-related protein A1, and BCL2-antagonist/killer 1) by RNAse protection assay (Figure 4b).

### Interferon response

**Figure 5:**
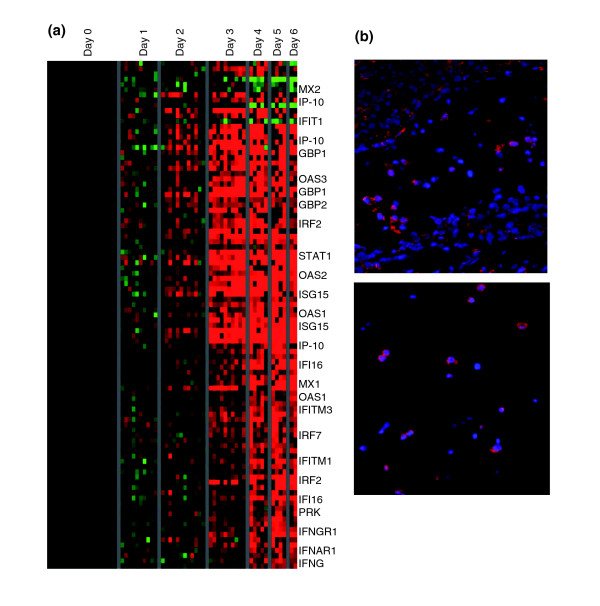
Interferon-responsive genes. **(a) **A list of known interferon (IFN) genes was compiled from the literature. The gene expression data for these genes was arranged by a self-organzing map, using ten nodes. **(b) **Myxovirus resistance protein (MX) expression in circulating cells. MX protein (red) was detected in circulating cells; cell nuclei are stained with DAPI (blue).

The earliest major transcriptional response apparent in all animals by day 2 or 3 was an increase in transcript levels of a large set of interferon (IFN) regulated genes (Figure 1), including the following: myxovirus resistance protein (MX)1 and MX2, IFN-γ inducible protein-10, 2'-5' oligoadenylate synthetase-1, -2, and -3, guanylate binding protein-1 and -2, signal transducer and activators of transcription (STAT)-1, double-stranded DNA activated protein kinase, and IFN-γ receptors 1 and 2. This response increased even further on day 4 and remained high throughout the time course of infection. We extracted the set of IFN regulated transcripts using previously published lists of known IFN-α, IFN-β, and IFN-γ induced genes [20,26,27] and arranged the gene expression data for these genes using a self-organzing map (Figure 5a). MX1 expression in circulating cells was confirmed by immunohistochemistry (Figure 5b).

### Fibrin deposition and dissolution

**Figure 6:**
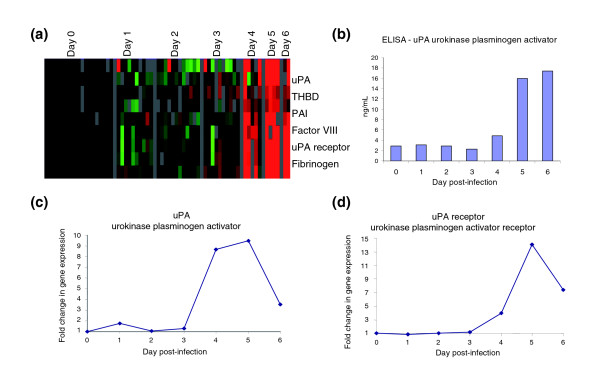
Fibrin deposition and dissolution. **(a) **Transcripts of genes known to be involved in the coagulation cascade (intrinsic and extrinsic pathways) were selected from the filtered dataset. Data were selected that showed a 2.5-fold change or greater in at least three arrays. **(b) **Protein levels of urokinase plasminogen activator (uPA) in blood plasma, as determined by ELISA. **(c and d) **Transcript levels of uPA (c) and uPA receptor (uPAR) (d).

Several transcripts related to the process of fibrin dissolution, including those for urokinase plasminogen activator (uPA) and uPA receptor, as well as the plasminogen activator inhibitor type 1 of the plasminogen-cleaving serine proteases, increased during days 4 to 6 after infection (Figure 6a,c,d). Expression of transcripts encoding uPA and uPA receptor rapidly increased from baseline on day 4 after infection and peaked on day 5 after infection (average fold above background: uPA, 9.5; uPA receptor, 14.1). uPA protein expression was confirmed by ELISA, and followed a similar trend as gene expression, but it continued to increase at day 6 after infection (Figure 6b).

### Proteolytic cleavage of the Ebola virus glycoprotein

**Figure 7:**
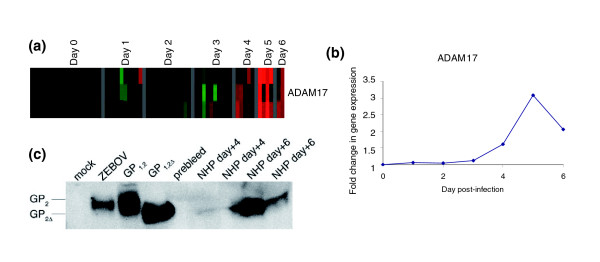
Expression levels of the metalloprotease responsible for cleavage of Ebola glycoprotein. Shown are **(a) **expression levels of tumor necrosis factor (TNF)-α converting enzyme/α-disintegrin and metalloproteinase (ADAM)-17 from the overview cluster and **(b) **in graph form. **(c) **Glycoprotein (GP) in the serum from infected rhesus macaques over the course of infection. Serum was diluted 1:3 in NP40 lysis buffer. Samples were run on a 10% Bis-Tris gel under reducing conditions, as shown. Mock cell lysate from 293T cells transfected with vector only (pDisplay) is shown as a negative control (lane 1); Zaire Ebola virus (ZEBOV; lane 2) is supernatant from *in vitro *Ebola infected Vero E6 cells at day 8 after infection. Lanes 3 and 4 are transfection controls expressing glycoprotein (GP)_1,2 _(cell lysate) and GP_1,2Δ _supernatant) [31]. Serum from infected rhesus macaques, before infection (lane 5), and on day 4 and 6 after infection (lanes 6 to 9) were diluted 1:3 in NP40 lysis buffer and 22.5 μl was loaded per lane. Samples included two animals per day (after infection) analyzed. Note the lack of GP in the prebleed control sample (lane 5). GP_2Δ _is seen in the transfection control (lane 4) and NHP sera samples from days 4 (lane 6, albeit weakly) and 6 days after infection (lanes 8 and 9).

We noted an increase in TNF-α converting enzyme/α-disintegrin and metalloproteinase (ADAM)-17 at days 4 to 6 after infection, peaking at an average 3.1-fold increase above baseline at day 5 after infection. Dolnik and coworkers [28] demonstrated that ADAM-17 is responsible for shedding of the EBOV glycoprotein (GP) ectodomain from cell surfaces *in vitro *[28]. We also detected the cleaved ectodomain of GP, GP_2Δ_, in sera from terminal (day 7 after infection) ZEBOV infected animals (Figure 7c), which was present at higher concentrations than the positive control of cell culture supernatant from ZEBOV infected Vero cells (Figure 7c).

### Pre-symptomatic transcriptional response in peripheral blood mononuclear cells

**Figure 8:**
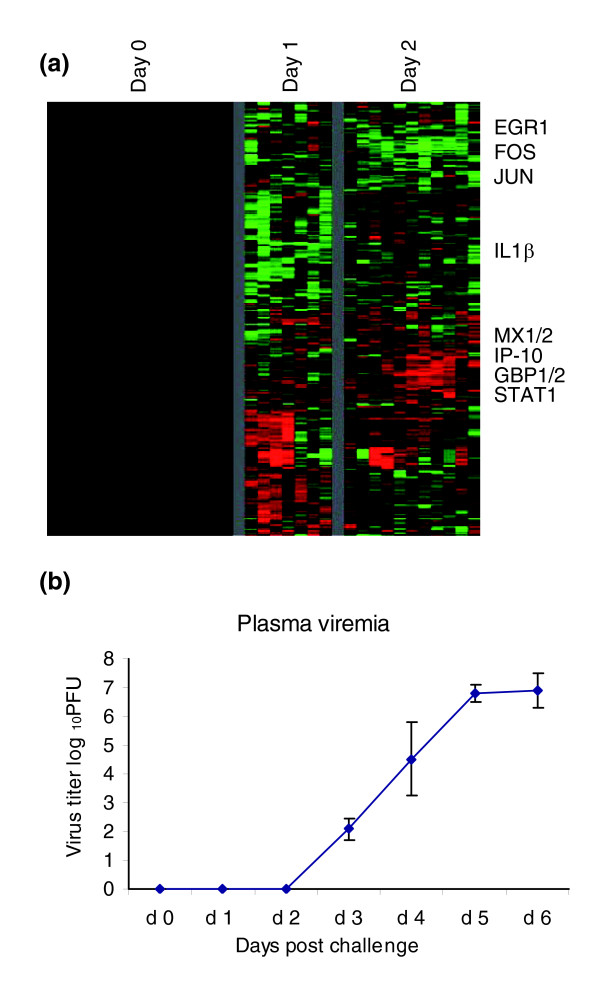
Preclinical gene expression. **(a) **Genes with transcripts whose abundance shows at least a twofold increase or decrease from baseline (day 0) in at least three of the samples for day 1 or day 2 are shown. The expression patterns of 317 elements (202 unique named genes) were hierarchically clustered; rows represent individual genes and columns represent samples. These patterns reflect changes in gene expression before symptoms appear. **(b) **Virus isolation from plasma. Infectious virus in EDTA plasma was assayed by counting plaqueson Vero cells maintained as monolayers in six-well plates under agarose, as previously described [70].

In order to determine whether we could detect gene expression changes before clinical symptoms appeared, we analyzed the complete dataset for genes that exhibited significant changes before day 3 after infection. The expression levels of 317 elements (202 unique named genes) either increased or decreased by at least twofold, in at least three animals, at day 1 or 2 after infection (Figure 8). IL-1β, which was highly induced at later stages of infection (Figure 2), was initially repressed on the first day after infection. Genes that were induced during the first 2 days after infection included early stress response genes (early growth response, Fos, Jun) and IFN responsive genes (MX1 and 2, STAT-1, IFN-γ inducible protein-10, guanylate binding protein-1 and -2). Animals had no detectable clinical illness at days 1 and 2, were feeding normally, had normal physical activity patterns on days 1 and 2, and normal results for all measured laboratory values (complete blood count, differential, chemistries, ELISA, and temperature). Levels of plasma viremia were undetectable until day 3 after infection (Figure 8b). In addition, there were only mild symptoms at day 3 after infection; three out of ten animals sampled had elevated temperature, and three out of 15 had early signs of rash (very mild) and a slight increase in D-dimers.

### Changes in cell component mixtures of peripheral blood mononuclear cells

In samples of whole blood or PBMCs, variations in the individual cell subtypes (lymphocytes, monocytes) that comprise the mixed cell population can lead to observed differences in gene expression responses. An increase or decrease in one cell type changes the overall proportion of that cell type's unique transcripts in the total pool of RNA from a given sample. To address this issue more effectively, we correlated the gene expression vector for each individual gene in the dataset with each parameter in the complete blood count and differential data on relative levels of individual cell populations (Additional data file 3). This allowed us to assess the magnitude of the contribution of changes in cell type to the observed gene expression profiles for each cluster. The largest average correlation scores for the two major clusters shown in Figure 1 were 0.45 (lymphocyte count, decreased transcript abundance cluster), 0.47 (total neutrophil count, increased transcript abundance), and 0.69 (band neutrophil count, increased transcript abundance).

## Discussion

In a series of studies we recently analyzed the pathology of lethal ZEBOV infection in cynomolgus macaques using a sequential sacrifice design [13,14]. In the present study, we examined the genome-wide transcriptional responses in sequential samples of peripheral blood from 15 of these cynomolgus macaques. Nonhuman primates infected with ZEBOV exhibited a highly homogeneous, time-dependent pattern of gene expression (Figure 1). Given the massive pathologic changes, physiologic instability, and widespread tissue damage, as well as the commonly observed variability in genome-wide transcript abundance patterns among different individual hosts *ex vivo*, it was surprising that the animals displayed such uniform patterns. Perhaps because of the overwhelming nature of the infection and the relatively short time frame between the first appearance of signs and death, these patterns are highly homogenous due to an effect akin to temporal compression. It is very likely that the observed gene expression patterns reflect many physiologic changes caused by systemic filoviral infection (for example, bystander lymphocyte apoptosis, fibrin deposition, and anti-viral IFN response). With a longer time frame or lower mortality rate, it is possible that individual host responses might show more variation; nonetheless, the homogeneity of this response allowed us to analyze the characteristic gene expression patterns with minimal noise from animal-to-animal variation. The underlying molecular changes echo the uniform lethality of the animal model, and may provide better predictors of morbidity/mortality than a model with high levels of inter-individual variation.

We observed a marked increase in transcript abundance for genes encoding many cytokines, including IL-1β, IL-6, IL-8, MIP-1α, MIP-1β, macrophage colony stimulating factor, and MCP-1 (Figure 2), which is consistent with a systemic proinflammatory response. Reports of cases of human EBOV infection vary considerably with respect to the cytokines that are associated with fatal as opposed to nonfatal outcome [12,25,29]. Increases in IL-1β, IL-6, MIP-1α, and MIP-1β have been reported for human survivors of EBOV infection [25]. *In vitro *infection of human monocytes/macrophages with authentic EBOV or virus-like particles that include membrane-associated GP_1,2 _leads to increases in protein levels of IL-1β [30-32], IL-6 [30-32], IL-8 [31,32], MIP-1α [30,33], MIP-1β [33], and MCP-1 [30]. In monkeys infected with ZEBOV or Reston Ebola virus, increases in IL-1β [14,34], IL-6 [14,33,34], MIP1-α [14,33], MIP-1β [14,33,34], and MCP-1 [14,34] have been reported. Monocytes and macrophages represent a major cellular target for infection and dissemination of EBOV in monkeys [14,35-37]. Infection of monocytes and macrophages leads to increased production and release of proinflammatory cytokines, leading in turn to recruitment of macrophages to areas of inflammation, which may contribute to viral proliferation and eventually an overwhelming sepsis-like syndrome [14,38,39].

Serum levels of TNF-α, in particular, are demonstrably increased in human [12,29], primate [14,33], and *in vitro *[30-33] EBOV infection. Wahl-Jensen and coworkers [40] recently showed that the virus-like particle induced decrease in endothelial barrier function was further enhanced by TNF-α, which is known to induce a long-lasting decrease in endothelial cell barrier function and is hypothesized to play a key role in EBOV pathogenesis [40]. We detected an increase not only in TNF-α but also in the downstream transcriptional response that is regulated by TNF-α and NF-κB (Figure 3), providing evidence that the circulating cells are responding to the large amounts of TNF-α that are induced during infection. Induction of the NF-κB pathway by TNF-α usually induces an anti-apoptotic response and cell survival [41], possibly reflecting a mechanism by which EBOV counteracts host apoptotic defenses in the infected cell, thereby contributing to viral spread.

Despite the known role of the NF-κB pathway in an anti-apoptotic response, we found that transcripts for many pro-apoptotic genes were induced (Figure 4). Genes for the Bcl antagonists BCL2-antagonist of cell death, BH3 interacting domain death agonist, BCL-X, and BAK appeared to be induced in the later stages of infection; all of these factors promote apoptosis by inhibiting Bcl-2. Expression levels of IL-1α and IL-1β were also increased; these cytokines are proteolytically processed and released in response to cell injury and induce apoptosis. Both forms of IL-1 are proteolytically processed to their active form by caspase 1, which was also expressed. In addition, transcript levels of TRAIL were markedly increased (Figure 4b). TRAIL expression early during infection and induction by IFN-α may contribute to lymphocyte apoptosis [33]. In view of the increased transcript levels for a group of pro-apoptotic genes, the decrease in lymphocyte related transcripts, including CD3, CD8, CD19, CD64, major histocompatibility complex class II, T cell receptor β, integrins, and granzymes (Figure 1) in ZEBOV infection may result from 'bystander' lymphocyte apoptosis and subsequent depletion of lymphocytes in circulating peripheral blood [14,23-25]. Thus, it appears that although the infected monocyte/macrophage lineages can survive and carry virus to secondary infection sites in the tissues, cells important for the adaptive immune response are decimated through bystander lymphocyte apoptosis, preventing an effective adaptive immune response, and enabling further virus propagation and spread.

Although the major transcriptional changes appeared on days 4 to 6, corresponding to the initial appearance of clinical signs, a strong IFN response was evident at day 3 after infection (Figure 5a), and transcripts levels for a subset of IFN genes increased as early as 24 hours after infection (Figure 8). In addition, expression of the classical IFN induced protein MxA was detected in circulating cells (Figure 5b). Several studies have reported the detection of IFN-α in serum from EBOV-infected humans [12] and monkeys [14,33], and our results provide evidence that cells in circulating peripheral blood can mount a robust transcriptional response to the IFN stimulus, despite the presence of EBOV proteins (VP24 and VP35), which are thought to function as type I IFN antagonists [42,43]. This might imply that the major role of the ZEBOV type I IFN antagonists is to act locally to influence the microenvironment of the infected cell, rather than to shut down a systemic IFN response. The majority of cells in the peripheral blood sample (PBMCs) are uninfected, because no evidence of EBOV infection of lymphocytes has been observed [14,23] and the circulating population of infected monocytes/macrophages constitutes only 1% to 13% of PBMCs in these primates. Both VP35 and VP24 act in a cell autonomous manner; VP35 blocks activation of the IFN regulatory factor 3 and the transcriptional responses of the IFN regulatory factor 3 responsive promoters [44], and VP24 blocks nuclear accumulation of tyrosine phosphorylated STAT through interaction with karyopherin α_1 _[43]. Because of the cell autonomous nature of the EBOV IFN antagonists, uninfected cells should still be capable of producing a transcriptional response to the large amounts of circulating IFN, as shown in Figure 5a.

Disseminated intravascular coagulation, caused by over-activation of the coagulation system and resulting in microvascular thrombosis [45], may contribute to the lethal multi-system organ failure in EBOV infection. Over-expression of tissue factor in EBOV infected monocytes/macrophages has been shown to produce fibrin deposition in the spleen, liver, and blood vessels of infected macaques [46], and inhibition of the tissue factor/factor VIIa pathway resulted in a decrease of D-dimers (fibrin degradation products) and an increased survival rate in rhesus macaques [47]. In this study we found evidence of cellular responses that would be expected to lead to increased fibrin degradation. There was an increase in both uPA and uPA receptor transcripts in PBMCs (Figure 6a,c,d), accompanied by an increase in serum concentrations of uPA protein (Figure 6b). uPA acts to convert plasminogen to plasmin; the uPA receptor mediates the proteolysis independent signal transduction activation effects of uPA, also promoting plasmin formation. However, we also observed an increase in transcripts encoding plasminogen activator inhibitor, perhaps caused by negative feedback regulation. Thus, the overall impact of the observed transcriptional response on the coagulation cascade is not self-evident. Nevertheless, although the majority of the coagulation and fibrinolytic cascade is regulated at the protein level through processing, transcriptional induction of genes that are involved in fibrin degradation may be a factor in the coagulopathy during EBOV infection.

EBOV GP is regulated by complex transcriptional editing and post-translational cleavage processes. The authentic transcript of the GP gene is expressed as a polypeptide, which is cleaved into soluble glycoprotein (sGP) and the secreted delta peptide [48,49]. Through RNA editing, the transmembrane form of GP is expressed (GP_1,2_) and then cleaved into GP_1 _and GP_2 _disulfide linked fragments, which are present on the surface of virus particles [50-52]. The role of EBOV GP and its contribution to pathogenesis has been the subject of much investigation. GP can decrease the expression of cell adhesion molecules, interfering with cell attachment and inducing cytotoxicity [53-56], but mutant viruses that fail to produce sGP are more cytotoxic, suggesting a negative regulation by sGP of the GP induced cytotoxicity [57]. *In vitro *studies suggest that GP_1,2 _on the surface of virus-like particles, but not sGP, activates target cells [31] and decreases endothelial barrier function [40]. However, EBOV replication does not induce direct cytolysis of endothelial cells either *in vitro *or in animal models of EBOV infection [13], although cytolytic infection of human umbilical cord vein endothelial cells has been demonstrated with Marburg virus [58].

TNF-α converting enzyme/ADAM-17 was recently found to mediate proteolytic processing and shedding of the ectodomain of Ebola GP (GP_1,2Δ_) [28]. We found that transcript levels for ADAM-17 increased on days 4 to 6 after infection, peaking at day 5 after infection (Figure 7a,b), which is consistent with a role for ADAM-17 in shedding of GP_1,2Δ _during *in vivo *primate infection. In addition, we also detected elevated concentrations of cleaved GP_2Δ _in sera from late-stage ZEBOV infected animals compared with uninfected controls (Figure 7c), demonstrating that cleavage of GP also takes place during *in vivo *infection in a nonhuman primate model of EBOV hemorrhagic fever. The relationship of shed GP_1,2Δ _to pathogenesis/disease severity is unclear, and its role during *in vivo *infection remains to be investigated. It is possible that GP_1,2Δ _can act as a decoy and soak up anti-EBOV antibodies, effectively shielding the virus from the immune system [28].

The composite gene expression pattern assayed in a mixed cell population, such as PBMCs, gives a rich and multidimensional picture of the systemic host responses to infection, reflecting many interconnected responses of a complex system. However, because the observed gene expression pattern represents a composite of diverse influences, data from mixed cell populations can be more difficult to interpret. In samples of whole blood or PBMCs, large variations in the cellular composition are often the largest source of overall variation in the observed gene expression patterns [59]. We correlated the gene expression vector for each individual gene in the dataset with complete blood count and differential data on relative levels of individual cell populations (Additional data file 3). Correlation scores were highest between cell populations that increased during the course of infection and the cluster of genes whose transcripts levels were increasing, and also cell populations that decreased and the cluster of genes whose transcripts levels were decreasing. As an example, the depletion of circulating lymphocytes during EBOV infection *in vivo *[24] correlates with the decrease in lymphocyte-related transcripts in our microarray dataset (Figure 1). However, given the mathematical simplicity of the gene expression and differential parameters (elements either increase or decrease uniformly and uniderectionally with respect to time), the correlation scores could be due to temporal coincidence of processes with similar directionality.

These correlations indicate that there is a certain proportion of the variation in gene expression that might be explained by changes in cell population. However, it is difficult to determine whether the variation in gene expression is directly caused by changes in the composition of the mixed cell population, activation of cells, or coincidence of temporal processes. Although correlative analysis based on numbers of PBMC types may provide the basis for attributing variation in expression to specific cell subsets, future experiments examining filovirus infection in more homogenous cell subpopulations will be essential for reliable identification of cell type specific responses. Likewise, examination of different tissues during the course of infection could provide a more comprehensive picture of the molecular anatomy of host responses to EBOV on an organism-wide basis.

Early detection and diagnosis of EBOV infection would be invaluable, as many of the symptoms and signs are nonspecific at presentation [2], and some recently described interventions have benefit when given early after filovirus infection [47,60]. To determine whether we could detect any changes in gene expression before appearance of symptoms, we examined the gene expression profile of peripheral blood in the early preclinical stages of infection (days 1 and 2). We observed changes in expression of over 200 genes before any clinical signs were evident and before plasma viremia was detectable in the ZEBOV infected nonhuman primate model (Figure 8a,b). This gene set is a possible starting point for the identification of early diagnostic markers. Any of these genes alone may provide little specificity, but combinations of these and/or others may allow differentiation among different etiologic agents, all of which may otherwise demonstrate similar clinical pictures. Although these early response genes are all responsive to diverse infections and inflammatory conditions and thus are of limited specificity for detection and diagnosis, the pattern of response to EBOV infection, in a setting with a high index of suspicion, may provide useful early warning for triage, aggressive treatment, and/or quarantine.

Analysis of global gene expression as disease pathogenesis unfolds provides a multifaceted picture of the complex interplay between host and pathogen. Examination of early events during infection may help to identify and provide insight into the specific molecular processes that initiate the cascade of host damage during EBOV infection. The ability to detect gene expression patterns before clinical symptoms may provide an opportunity for early diagnosis.

## Materials and methods

### Nonhuman primate model of Ebola infection

Fifteen healthy, adult male cynomolgus macaques were inoculated intramuscularly in the left or right caudal thigh with 1,000 plaque forming units of ZEBOV [61]. Animals were killed on days 1, 2, 3, 4, 5, and 6 after infection [13,14,46]. Infection studies were performed under biosafety level 4 containment at the US Army Medical Research Institute of Infectious Diseases. Research was conducted in compliance with the Animal Welfare Act and other federal statues and regulations relating to animals and experiments involving animals, and adheres to the principles stated in the Guide for the Care and Use of Laboratory Animals (National Research Council, 1996). The biosafety level 4 facility used is fully accredited by the Association for Assessment and Accreditation of Laboratory Animal Care International.

### Sample acquisition and RNA preparation

Peripheral blood samples (2.5 ml) were collected on days 1, 4, and 6 before infection, in order to define a robust baseline, and then on successive days after infection until death (immediately before their death) or recovery. All samples were collected at the same time of day (± 2 hours) to minimize differences in expression caused by diurnal variation. PBMCs were isolated from 1.5 ml of peripheral blood using by centrifugation on Histopaque (Sigma, St. Louis, MO, USA) at 250 *g *for 30 min. Cells at the interface were harvested, washed twice in phosphate-buffered saline, and placed in TRIzol (Invitrogen Corporation, Carlsbad, CA, USA). Total RNA was extracted using TRIzol. RNA was linearly amplified using the Ambion MessageAmp kit (Ambion Inc, Austin, TX, USA).

### cDNA microarrays and hybridization

We used human cDNA microarrays containing 37,632 elements that represent approximately 18,000 unique genes, which efficiently capture monkey transcripts [20]. Arrays were produced as described previously [62-64]. Fluorescently labeled cDNA prepared from amplified RNA was hybridized to the array in a two-color comparative format [62,65], with the experimental samples labeled with one fluorophore (Cy5) and a reference pool of mRNA labeled with a second fluorophore (Cy3). The reference pool (Universal Human Reference; Stratagene Inc., La Jolla, CA, USA) provided an internal standard to enable reliable comparison of relative transcript levels in multiple samples [62,63,66]. The microarrays were submitted to the Gene Expression Omnibus database under series record GSE8317.

### Data filtering and analysis

Array images were scanned using an Axon Scanner 4000A (Axon Instruments, Union City, CA, USA), and image analysis was performed using GenePix Pro version 3.0.6.89 (Axon Instruments). Data were expressed as the log_2 _ratio of fluorescence intensities of the sample and the reference, for each element on the array [62,65]. Data were filtered to exclude elements that did not have a regression correlation of Cy5 to Cy3 signal over the pixels spanning the array element of ≥0.6 and intensity/background ratio of ≥2.5 in at least 80% of the arrays. For each gene, the expression levels over the time course for each monkey were 'time zero-transformed' by subtracting the average of the pre-infection baseline expression level from that animal, so that the values of each time point represent changes relative to the uninfected samples. The genes whose expression varied from the uninfected baseline by at least threefold in at least three samples were selected for further analysis. There was relatively little variation in the pre-infection baseline samples; in the 39 pre-exposure samples only 769 elements (439 unique named genes) varied at least threefold in at least three samples, as compared with 3,670 elements (1,832 named genes) that varied at least threefold in three post-exposure samples. The data were hierarchically clustered using the Cluster program [67] and displayed using TreeView [68].

### Hematology

Total white blood cell counts, lymphocyte counts, red blood cell counts, platelet counts, hematocrit values, total hemoglobin, mean cell volume, mean corpuscular volume, and mean corpuscular hemoglobin concentration were determined from blood samples collected in tubes containing EDTA, using a laser-based hematological Analyzer (Coulter Electronics, Hialeah, FL, USA). White blood cell differentials were measured manually on Wright-stained blood smears.

### Cytokine and chemokine ELISAs

Cytokine/chemokine levels in monkey sera/plasma were assayed using commercially available ELISA kits according to manufacturer's directions. Cytokines/chemokines assayed included monkey TNF-α (BioSource International, Inc., Camarillo, CA, USA). ELISAs for human proteins known to be compatible with cynomolgus macaques included IL-6, MIP-1α, and MIP-1β (BioSource International, Inc.), and human IL-18 and MCP-1 (R&D Systems, Minneapolis, MN, USA).

### RNase protection assays

PBMCs were prepared through a Histopaque gradient as described above, washed in RPMI 1640, and placed in TRIzol. The Multiprobe RNase Protection Assay was performed in accordance with the manufacturer's directions (Pharmingen, San Diego, CA, USA) with minor modifications as described previously [33].

### Immunofluorescence

De-paraffinized tissue sections were pretreated with proteinase K (20 μg/ml; DAKO, Carpinteria, CA, USA) for 30 min at room temperature and incubated in normal goat serum for 20 minutes (DAKO). Sections were then incubated with an anti-MxA antibody (mouse monoclonal antibody M143 directed against a conserved epitope in the amino-terminal half of the MxA molecule [69], courtesy of Otto Haller) for 30 min at room temperature. After incubation, sections were placed in Alexa Fluor^® ^594 goat anti-mouse IgG_1 _(Molecular Probes, Carlsbad, CA, USA) for 30 minutes at room temperature and rinsed. After rinsing in phosphate-buffered saline, sections were mounted in an aqueous mounting medium containing 4',6'-diamidino-2-phenylindole (Vector Laboratories, Burlingame, CA, USA) and examined with a Nikon E600 fluorescence microscope (Nikon Instech Co., Ltd., Kanagawa, Japan).

### Western blotting for truncated glycoprotein 2 (GP_2Δ_) in ZEBOV infected animals

Monkey sera were diluted 1:3 in NP-40 lysis buffer (10 mmol/l Tris [pH 7.5], 3% 5 mol/l NaCl, 1% NP40 and complete protease inhibitor tablet [Roche Applied Science, Indianapolis, IN, USA]). Controls included ZEBOV seed stock diluted 1:3 with NP40 lysis buffer, and glycoprotein controls were generated by transfecting 293T cells with GP_1,2 _or GP_1,2Δ _plasmids, as described previously [31]. Cells and supernatants were harvested at 48 hours after transfection. Samples were diluted in NuPage LDS sample buffer and NuPage sample reducing agent (Invitrogen Corporation), boiled and then loaded on a 10% Bis-Tris acrylamide gel, and run using NuPage MES buffer (Invitrogen Corporation). Samples were run with a SeeBlue Plus 2 standard (Invitrogen Corporation). The gel was transferred to a nitrocellulose membrane and blocked overnight at 4°C with Tris-buffered saline with 0.05% Tween-20 (TBS-T) containing 10% milk. The membrane was washed three times with TBS-T for 5 min per wash. Following these washes, membranes were incubated with the primary anti-EBOV glycoprotein (GP_2Δ_) antibody (1:500) for 2 hours at room temperature (rabbit anti-GP_2 _IgG [28]; antibody kindly provided by V Volchkov, Lyon, France). Membranes were washed three times for 5 min with TBS-T then incubated with the secondary anti-rabbit IgG horseradish peoxidase antibody (1:30,000) at room temperature. Following secondary antibody incubation, the membranes were washed twice for 5 min with TBS-T and three times for 5 min with Tris-buffered saline only, and analyzed using the SuperSignal West Femto maximum sensitivity chemiluminescent substrate following the manufacturer's instructions (Pierce, Rockford, IL, USA).

## Abbreviations

ADAM, α-disintegrin and metalloproteinase; BCL-X, BCL2-like 1; EBOV, Ebola virus; ELISA, enzyme-linked immunosorbent assay; GP, glycoprotein; IFN, interferon; IL, interleukin; MCP, monocyte chemoattractant protein; MIP, macrophage inflammatory protein; MX, myxovirus resistance protein; NF-κB, nuclear factor-κB; PBMC, peripheral blood mononuclear cell; sGP, soluble glycoprotein; STAT, signal transducer and activators of transcription; TBS-T, Tris-buffered saline with 0.05% Tween-20; TNF, tumor necrosis factor; TRAIL, TNF related apoptosis inducing ligand; uPA, urokinase plasminogen activator; ZEBOV, Zaire Ebola virus.

## Authors' contributions

KHR conceived, designed, and executed the experiments described in this report and wrote the manuscript. LEH and TWG designed and performed the animal studies. VJ and KMD performed the Western blot experiment. HAY performed the RNase protection assays. POB, DAR, LEH and TWG oversaw completion of the studies as well as the final manuscript. All authors read and approved the final version of the manuscript.

## Additional data files

The following additional data are available with the online version of this paper. Additional data file 1 provides animal numbers for blood samples. Additional data file 2 shows the bleed schedule. Additional data file 3 shows the gene expression correlation with immune cell types.

## Supplementary Material

Additional data file 1 Animal tattoo number for each blood sample listed by Day. Gene expression profiles in all figures are arranged from left to right for each day post-infection, as listed in the table. Click here for file

Additional data file 2 Each bleed day for each animal is indicated with an X. Serial samples for all animals on all days were not taken, due to Laboratory Animal care and Use Committee restrictions on maximum blood volume amounts. Click here for file

Additional data file 3 Correlation coefficients were calculated between the expression pattern of each gene and each clinical parameter. The correlation coefficients are plotted as moving averages of 41 genes. Click here for file

## References

[B1] Feldmann H, Geisbert TW, Jahrling PB, Klenk HD, Netesov SV, Peters CJ, Sanchez A, Swanepoel R, Volchkov VE, Fauquet CM, Mayo MA, Maniloff J, Desselberger U, Ball LA (2005). Filoviridae.. Virus Taxonomy: VIIIth Report of the International Committee on Taxonomy of Viruses.

[B2] Sanchez A, Kahn AS, ZS R, Nabel GL, Ksiazek TG, Peters CJ, Knipe DM, Howley PM (2001). Filoviridae.. Fields Virology.

[B3] Jahrling PB, Geisbert TW, Dalgard DW, Johnson ED, Ksiazek TG, Hall WC, Peters CJ (1990). Preliminary report: isolation of Ebola virus from monkeys imported to USA.. Lancet.

[B4] Jahrling PB, Geisbert TW, Jaax NK, Hanes MA, Ksiazek TG, Peters CJ (1996). Experimental infection of cynomolgus macaques with Ebola-Reston filoviruses from the 1989-1990 U.S. epizootic.. Arch Virol Suppl.

[B5] Dalgard DW, Hardy RJ, Pearson SL, Pucak GJ, Quander RV, Zack PM, Peters CJ, Jahrling PB (1992). Combined simian hemorrhagic fever and Ebola virus infection in cynomolgus monkeys.. Lab Anim Sci.

[B6] Le Guenno B, Formentry P, Wyers M, Gounon P, Walker F, Boesch C (1995). Isolation and partial characterisation of a new strain of Ebola virus.. Lancet.

[B7] Ebola infection in Côte d'Ivoire/Liberia. http://www.who.int/csr/don/1996_01_22c/en/index.html.

[B8] Borio L, Inglesby T, Peters CJ, Schmaljohn AL, Hughes JM, Jahrling PB, Ksiazek T, Johnson KM, Meyerhoff A, O'Toole T (2002). Hemorrhagic fever viruses as biological weapons: medical and public health management.. JAMA.

[B9] Stroher U, Feldmann H (2006). Progress towards the treatment of Ebola haemorrhagic fever.. Expert Opin Investig Drugs.

[B10] Hensley LE, Jones SM, Feldmann H, Jahrling PB, Geisbert TW (2005). Ebola and Marburg viruses: pathogenesis and development of countermeasures.. Curr Mol Med.

[B11] Feldmann H, Jones SM, Schnittler HJ, Geisbert T (2005). Therapy and prophylaxis of Ebola virus infections.. Curr Opin Investig Drugs.

[B12] Villinger F, Rollin PE, Brar SS, Chikkala NF, Winter J, Sundstrom JB, Zaki SR, Swanepoel R, Ansari AA, Peters CJ (1999). Markedly elevated levels of interferon (IFN)-gamma, IFN-alpha, interleukin (IL)-2, IL-10, and tumor necrosis factor-alpha associated with fatal Ebola virus infection.. J Infect Dis.

[B13] Geisbert TW, Young HA, Jahrling PB, Davis KJ, Larsen T, Kagan E, Hensley LE (2003). Pathogenesis of Ebola hemorrhagic fever in primate models: evidence that hemorrhage is not a direct effect of virus-induced cytolysis of endothelial cells.. Am J Pathol.

[B14] Geisbert TW, Hensley LE, Larsen T, Young HA, Reed DS, Geisbert JB, Scott DP, Kagan E, Jahrling PB, Davis KJ (2003). Pathogenesis of Ebola hemorrhagic fever in cynomolgus macaques: evidence that dendritic cells are early and sustained targets of infection.. Am J Pathol.

[B15] Zaki SR, Goldsmith CS (1999). Pathologic features of filovirus infections in humans.. Curr Top Microbiol Immunol.

[B16] Jahrling PB, Geisbert J, Swearengen JR, Jaax GP, Lewis T, Huggins JW, Schmidt JJ, LeDuc JW, Peters CJ (1996). Passive immunization of Ebola virus-infected cynomolgus monkeys with immunoglobulin from hyperimmune horses.. Arch Virol Suppl.

[B17] Fisher-Hoch SP, Brammer TL, Trappier SG, Hutwagner LC, Farrar BB, Ruo SL, Brown BG, Hermann LM, Perez-Oronoz GI, Goldsmith CS (1992). Pathogenic potential of filoviruses: role of geographic origin of primate host and virus strain.. J Infect Dis.

[B18] Geisbert TW, Pushko P, Anderson K, Smith J, Davis KJ, Jahrling PB (2002). Evaluation in nonhuman primates of vaccines against Ebola virus.. Emerg Infect Dis.

[B19] Sullivan NJ, Sanchez A, Rollin PE, Yang ZY, Nabel GJ (2000). Development of a preventive vaccine for Ebola virus infection in primates.. Nature.

[B20] Rubins KH, Hensley LE, Jahrling PB, Whitney AR, Geisbert TW, Huggins JW, Owen A, Leduc JW, Brown PO, Relman DA (2004). The host response to smallpox: analysis of the gene expression program in peripheral blood cells in a nonhuman primate model.. Proc Natl Acad Sci USA.

[B21] Jahrling PB, Hensley LE, Martinez MJ, Leduc JW, Rubins KH, Relman DA, Huggins JW (2004). Exploring the potential of variola virus infection of cynomolgus macaques as a model for human smallpox.. Proc Natl Acad Sci USA.

[B22] Boldrick JC, Alizadeh AA, Diehn M, Dudoit S, Liu CL, Belcher CE, Botstein D, Staudt LM, Brown PO, Relman DA (2002). Stereotyped and specific gene expression programs in human innate immune responses to bacteria.. Proc Natl Acad Sci USA.

[B23] Geisbert TW, Hensley LE, Gibb TR, Steele KE, Jaax NK, Jahrling PB (2000). Apoptosis induced in vitro and in vivo during infection by Ebola and Marburg viruses.. Lab Invest.

[B24] Reed DS, Hensley LE, Geisbert JB, Jahrling PB, Geisbert TW (2004). Depletion of peripheral blood T lymphocytes and NK cells during the course of ebola hemorrhagic Fever in cynomolgus macaques.. Viral Immunol.

[B25] Baize S, Leroy EM, Georges-Courbot MC, Capron M, Lansoud-Soukate J, Debre P, Fisher-Hoch SP, McCormick JB, Georges AJ (1999). Defective humoral responses and extensive intravascular apoptosis are associated with fatal outcome in Ebola virus-infected patients.. Nat Med.

[B26] Der SD, Zhou A, Williams BR, Silverman RH (1998). Identification of genes differentially regulated by interferon alpha, beta, or gamma using oligonucleotide arrays.. Proc Natl Acad Sci USA.

[B27] Boehm U, Klamp T, Groot M, Howard JC (1997). Cellular responses to interferon-gamma.. Annu Rev Immunol.

[B28] Dolnik O, Volchkova V, Garten W, Carbonnelle C, Becker S, Kahnt J, Stroher U, Klenk HD, Volchkov V (2004). Ectodomain shedding of the glycoprotein GP of Ebola virus.. EMBO J.

[B29] Baize S, Leroy EM, Georges AJ, Georges-Courbot MC, Capron M, Bedjabaga I, Lansoud-Soukate J, Mavoungou E (2002). Inflammatory responses in Ebola virus-infected patients.. Clin Exp Immunol.

[B30] Gupta M, Mahanty S, Ahmed R, Rollin PE (2001). Monocyte-derived human macrophages and peripheral blood mononuclear cells infected with ebola virus secrete MIP-1alpha and TNF-alpha and inhibit poly-IC-induced IFN-alpha in vitro.. Virology.

[B31] Wahl-Jensen V, Kurz SK, Hazelton PR, Schnittler HJ, Stroher U, Burton DR, Feldmann H (2005). Role of Ebola virus secreted glycoproteins and virus-like particles in activation of human macrophages.. J Virol.

[B32] Stroher U, West E, Bugany H, Klenk HD, Schnittler HJ, Feldmann H (2001). Infection and activation of monocytes by Marburg and Ebola viruses.. J Virol.

[B33] Hensley LE, Young HA, Jahrling PB, Geisbert TW (2002). Proinflammatory response during Ebola virus infection of primate models: possible involvement of the tumor necrosis factor receptor superfamily.. Immunol Lett.

[B34] Hutchinson KL, Villinger F, Miranda ME, Ksiazek TG, Peters CJ, Rollin PE (2001). Multiplex analysis of cytokines in the blood of cynomolgus macaques naturally infected with Ebola virus (Reston serotype).. J Med Virol.

[B35] Davis KJ, Anderson AO, Geisbert TW, Steele KE, Geisbert JB, Vogel P, Connolly BM, Huggins JW, Jahrling PB, Jaax NK (1997). Pathology of experimental Ebola virus infection in African green monkeys. Involvement of fibroblastic reticular cells.. Arch Pathol Lab Med.

[B36] Jaax NK, Davis KJ, Geisbert TJ, Vogel P, Jaax GP, Topper M, Jahrling PB (1996). Lethal experimental infection of rhesus monkeys with Ebola-Zaire (Mayinga) virus by the oral and conjunctival route of exposure.. Arch Pathol Lab Med.

[B37] Geisbert TW, Jahrling PB, Hanes MA, Zack PM (1992). Association of Ebola-related Reston virus particles and antigen with tissue lesions of monkeys imported to the United States.. J Comp Pathol.

[B38] Schnittler HJ, Feldmann H (1998). Marburg and Ebola hemorrhagic fevers: does the primary course of infection depend on the accessibility of organ-specific macrophages?. Clin Infect Dis.

[B39] Bray M, Mahanty S (2003). Ebola hemorrhagic fever and septic shock.. J Infect Dis.

[B40] Wahl-Jensen VM, Afanasieva TA, Seebach J, Stroher U, Feldmann H, Schnittler HJ (2005). Effects of Ebola virus glycoproteins on endothelial cell activation and barrier function.. J Virol.

[B41] Wallach D, Varfolomeev EE, Malinin NL, Goltsev YV, Kovalenko AV, Boldin MP (1999). Tumor necrosis factor receptor and Fas signaling mechanisms.. Annu Rev Immunol.

[B42] Basler CF, Wang X, Muhlberger E, Volchkov V, Paragas J, Klenk HD, Garcia-Sastre A, Palese P (2000). The Ebola virus VP35 protein functions as a type I IFN antagonist.. Proc Natl Acad Sci USA.

[B43] Reid SP, Leung LW, Hartman AL, Martinez O, Shaw ML, Carbonnelle C, Volchkov VE, Nichol ST, Basler CF (2006). Ebola virus VP24 binds karyopherin a1 and blocks STAT1 nuclear accumulation.. J Virol.

[B44] Basler CF, Mikulasova A, Martinez-Sobrido L, Paragas J, Muhlberger E, Bray M, Klenk HD, Palese P, Garcia-Sastre A (2003). The Ebola virus VP35 protein inhibits activation of interferon regulatory factor 3.. J Virol.

[B45] Mammen EF (2000). Disseminated intravascular coagulation (DIC).. Clin Lab Sci.

[B46] Geisbert TW, Young HA, Jahrling PB, Davis KJ, Kagan E, Hensley LE (2003). Mechanisms underlying coagulation abnormalities in ebola hemorrhagic fever: overexpression of tissue factor in primate monocytes/macrophages is a key event.. J Infect Dis.

[B47] Geisbert TW, Hensley LE, Jahrling PB, Larsen T, Geisbert JB, Paragas J, Young HA, Fredeking TM, Rote WE, Vlasuk GP (2003). Treatment of Ebola virus infection with a recombinant inhibitor of factor VIIa/tissue factor: a study in rhesus monkeys.. Lancet.

[B48] Volchkova VA, Klenk HD, Volchkov VE (1999). Delta-peptide is the carboxy-terminal cleavage fragment of the nonstructural small glycoprotein sGP of Ebola virus.. Virology.

[B49] Volchkova VA, Feldmann H, Klenk HD, Volchkov VE (1998). The nonstructural small glycoprotein sGP of Ebola virus is secreted as an antiparallel-orientated homodimer.. Virology.

[B50] Sanchez A, Trappier SG, Mahy BW, Peters CJ, Nichol ST (1996). The virion glycoproteins of Ebola viruses are encoded in two reading frames and are expressed through transcriptional editing.. Proc Natl Acad Sci USA.

[B51] Volchkov VE, Becker S, Volchkova VA, Ternovoj VA, Kotov AN, Netesov SV, Klenk HD (1995). GP mRNA of Ebola virus is edited by the Ebola virus polymerase and by T7 and vaccinia virus polymerases.. Virology.

[B52] Volchkov VE, Feldmann H, Volchkova VA, Klenk HD (1998). Processing of the Ebola virus glycoprotein by the proprotein convertase furin.. Proc Natl Acad Sci USA.

[B53] Sullivan NJ, Peterson M, Yang ZY, Kong WP, Duckers H, Nabel E, Nabel GJ (2005). Ebola virus glycoprotein toxicity is mediated by a dynamin-dependent protein-trafficking pathway.. J Virol.

[B54] Simmons G, Wool-Lewis RJ, Baribaud F, Netter RC, Bates P (2002). Ebola virus glycoproteins induce global surface protein down-modulation and loss of cell adherence.. J Virol.

[B55] Chan SY, Ma MC, Goldsmith MA (2000). Differential induction of cellular detachment by envelope glycoproteins of Marburg and Ebola (Zaire) viruses.. J Gen Virol.

[B56] Yang ZY, Duckers HJ, Sullivan NJ, Sanchez A, Nabel EG, Nabel GJ (2000). Identification of the Ebola virus glycoprotein as the main viral determinant of vascular cell cytotoxicity and injury.. Nat Med.

[B57] Volchkov VE, Volchkova VA, Muhlberger E, Kolesnikova LV, Weik M, Dolnik O, Klenk HD (2001). Recovery of infectious Ebola virus from complementary DNA: RNA editing of the GP gene and viral cytotoxicity.. Science.

[B58] Schnittler HJ, Mahner F, Drenckhahn D, Klenk HD, Feldmann H (1993). Replication of Marburg virus in human endothelial cells. A possible mechanism for the development of viral hemorrhagic disease.. J Clin Invest.

[B59] Liu M, Popper SJ, Rubins KH, Relman DA (2006). Early days: genomics and human responses to infection.. Curr Opin Microbiol.

[B60] Daddario-DiCaprio KM, Geisbert TW, Stroher U, Geisbert JB, Grolla A, Fritz EA, Fernando L, Kagan E, Jahrling PB, Hensley LE (2006). Postexposure protection against Marburg haemorrhagic fever with recombinant vesicular stomatitis virus vectors in non-human primates: an efficacy assessment.. Lancet.

[B61] Jahrling PB, Geisbert J, Swearengen JR, Jaax GP, Lewis T, Huggins JW, Schmidt JJ, LeDuc JW, Peters CJ (1996). Passive immunization of Ebola virus-infected cynomolgus monkeys with immunoglobulin from hyperimmune horses.. Arch Virol.

[B62] Alizadeh AA, Eisen MB, Davis RE, Ma C, Lossos IS, Rosenwald A, Boldrick JC, Sabet H, Tran T, Yu X (2000). Distinct types of diffuse large B-cell lymphoma identified by gene expression profiling.. Nature.

[B63] Alizadeh A, Eisen M, Davis RE, Ma C, Sabet H, Tran T, Powell JI, Yang L, Marti GE, Moore DT (1999). The lymphochip: a specialized cDNA microarray for the genomic-scale analysis of gene expression in normal and malignant lymphocytes.. Cold Spring Harb Symp Quant Biol.

[B64] Brown Lab Protocols. http://cmgm.stanford.edu/pbrown/protocols/index.html.

[B65] Eisen MB, Brown PO (1999). DNA arrays for analysis of gene expression.. Methods Enzymol.

[B66] Perou CM, Sorlie T, Eisen MB, van de Rijn M, Jeffrey SS, Rees CA, Pollack JR, Ross DT, Johnsen H, Akslen LA (2000). Molecular portraits of human breast tumours.. Nature.

[B67] Eisen MB, Spellman PT, Brown PO, Botstein D (1998). Cluster analysis and display of genome-wide expression patterns.. Proc Natl Acad Sci USA.

[B68] Saldanha AJ (2004). Java Treeview: extensible visualization of microarray data.. Bioinformatics.

[B69] Flohr F, Schneider-Schaulies S, Haller O, Kochs G (1999). The central interactive region of human MxA GTPase is involved in GTPase activation and interaction with viral target structures.. FEBS Lett.

[B70] Jahrling PB, Murray PR, Baron EJ, Pfaller M, Tenover FC, Yolken RH (1999). Filoviruses and arenaviruses.. Manual of Clinical Microbiology.

