# Supplementary material for: The temporal program of peripheral blood gene expression in the response of nonhuman primates to Ebola hemorrhagic fever
Source: Genome Biol. 2007 Aug 28;8(8):R174. doi: 10.1186/gb-2007-8-8-r174 (PMC2375004; doi:10.1186/gb-2007-8-8-r174)
Supplement: Additional data file 1 — Animal tattoo number for each blood sample listed by Day. Gene expression profiles in all figures are arranged from left to right for each day post-infection, as listed in the table. [file gb-2007-8-8-r174-S1.pdf]

Day 0	Day 1	Day 2	Day 3	Day 4	Day 5	Day 6
28-221	331	28-221	28-332	9108	28-221	28-332
28-332	717	28-332	323	9878	323	9878
323	8667	323	32q		359	
32q	9028	331	359		48-143	
331	9093	359	8667			
359	9108	48-143	9093			
48-143	9877	8667	9108			
717		9028				
8667		9112				
9028		9878				
9093		9093				
9108						
9112						
9877						
9878						
