# Supplementary material for: The temporal program of peripheral blood gene expression in the response of nonhuman primates to Ebola hemorrhagic fever
Source: Genome Biol. 2007 Aug 28;8(8):R174. doi: 10.1186/gb-2007-8-8-r174 (PMC2375004; doi:10.1186/gb-2007-8-8-r174)
Supplement: Additional data file 2 — Each bleed day for each animal is indicated with an X. Serial samples for all animals on all days were not taken, due to Laboratory Animal care and Use Committee restrictions on maximum blood volume amounts. [file gb-2007-8-8-r174-S2.pdf]

| | Day 0 | Day 1 | Day 2 | Day 3 | Day 4 | Day 5 | Day 6 |
|---|---|---|---|---|---|---|---|
| **28-221** | x | | x | x | | x | |
| **28-332** | x | | x | x | | | x |
| **323** | x | | x | x | | x | |
| **32q** | x | | | | | | |
| **331** | x | x | x | x | | | |
| **359** | x | | x | x | | x | |
| **48-143** | x | | x | x | | x | |
| **717** | x | x | | | | | |
| **8667** | x | x | x | x | | | |
| **9028** | x | x | x | x | | | |
| **9093** | x | x | | x | | | |
| **9108** | x | x | | | x | | |
| **9112** | x | | x | x | | | |
| **9877** | x | x | | | | | |
| **9878** | x | | x | x | x | | x |
