# Supplementary figures and images for: The temporal program of peripheral blood gene expression in the response of nonhuman primates to Ebola hemorrhagic fever
Source: Genome Biol. 2007 Aug 28;8(8):R174. doi: 10.1186/gb-2007-8-8-r174 (PMC2375004; doi:10.1186/gb-2007-8-8-r174)

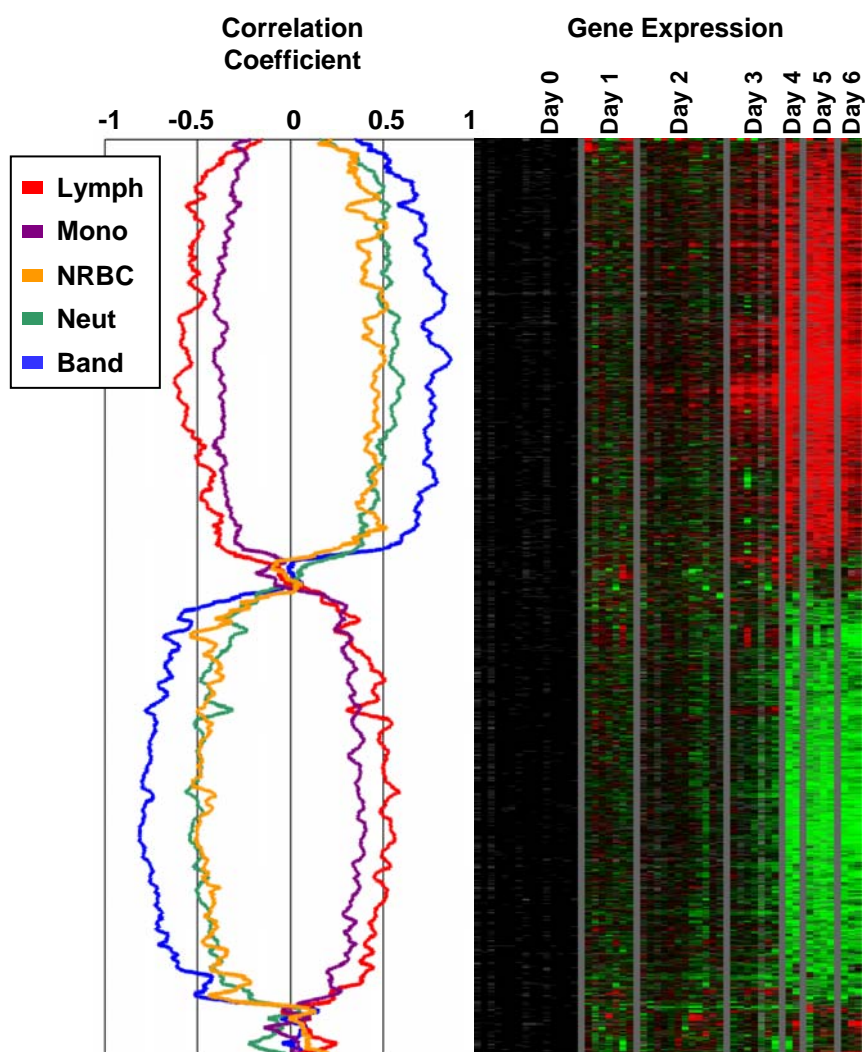

Supplement: Additional data file 3 — Correlation coefficients were calculated between the expression pattern of each gene and each clinical parameter. The correlation coefficients are plotted as moving averages of 41 genes. [file gb-2007-8-8-r174-S3.pdf]
